# Two new lichenized species and a new record from Guizhou, China

**DOI:** 10.3897/mycokeys.128.170469

**Published:** 2026-02-04

**Authors:** Lin-Zhi He, Wei Wu, He-Yun Bo, Lin-Shan Chai, Ruvishika S. Jayawardena, Sheng Liang, Qing-Feng Meng, Shao-Bin Fu

**Affiliations:** 1 School of Pharmacy, Zunyi Medical University, Zunyi, Guizhou Province 563000, China Zunyi Medical University Zunyi China; 2 Center of Excellence in Fungal Research, Mae Fah Luang University, Chiang Rai 57100, Thailand School of Public Health, Zunyi Medical University Zunyi China; 3 School of Science, Mae Fah Luang University, Chiang Rai 57100, Thailand Center of Excellence in Fungal Research, Mae Fah Luang University Chiang Rai Thailand; 4 Chishui Alsophila National Nature Reserve, Zunyi, Guizhou Province 564704, China School of Science, Mae Fah Luang University Chiang Rai Thailand; 5 School of Public Health, Zunyi Medical University, Zunyi, Guizhou Province 563000, China Chishui Alsophila National Nature Reserve Zunyi China

**Keywords:** 2 new species, geographical record, lichenized fungi, morphology, phylogeny, taxonomy

## Abstract

Two new lichenized species, *Chicitaea yueliangshanensis* and *Coniocarpon chishuiense* are described through the combination of morphological characteristics, chemical profiling and phylogenetic analyses, along with a new geographical record of *Synarthonia inconspicua*, collected from Guizhou, China. *Chicitaea yueliangshanensis* is characterized by prominent lecanorine apothecia with a black, epruinose, ± flat disc and lacking isidia and soredia. This species contains perlatolic acid, 2’-O-methylperlatolic acid and an unidentified lichen substance. It forms a well-supported singleton based on both ML and Bayesian analyses in the phylogenetic tree. Another new species, *Coniocarpon chishuiense* is distinguished by irregularly rounded to elliptical ascomata with epruinose disc and the presence of psoromic acid along with an unknown compound. Detailed morphological descriptions, illustrations and a compiled species checklist summarizing diagnostic features are provided.

## Introduction

The lichenized fungal genus *Chicitaea* Guzow-Krzem., Kukwa & Lendemer is a recently segregated group of lichenized fungi within the order Sarrameanales (Lecanoromycetes, Ascomycota), established based on combined morphological, chemical, and molecular evidence (Ptach-Styn et al. 2024). Species in this genus are characterized by a crustose thallus, the production of 2'-O-methylperlatolic acid as a major secondary metabolite, and apothecia with simple asci lacking an amyloid apical dome and bearing hyaline, non-septate ascospores (Lumbsch et al. 2007; Papong et al. 2009). Four species have been formally transferred into *Chicitaea*, i.e., *C. assateaguensis* (Lendemer) Guzow-Krzem., Kukwa & Lendemer, *C. confusa* (Lendemer) Guzow-Krzem., Kukwa & Lendemer, *C. cristinae* (Guzow-Krzem., Łubek, Kubiak & Kukwa) Guzow-Krzem., Kukwa & Lendemer, and *C. lecanoriformis* (Lumbsch, A.W. Archer & Elix) Guzow-Krzem., Kukwa & Lendemer (Ptach-Styn et al. 2024).

*Chicitaea* was previously included within *Loxospora* A. Massal. (Ascomycota, Lecanoromycetes, Sarrameanales, Sarrameanaceae). In *Loxospora* sensu stricto, asci typically possess a prominently developed dome-shaped tholus that reacts uniform amyloid, hyaline ascospores predominantly non-septate or with rudimentary septation, and display a helical twisting morphology. Paraphyses are simple to moderately branched. Phylogenetic studies have demonstrated that *Loxospora* comprised two distinct clades: one containing species with thamnolic acid and amyloid asci (*Loxospora* s. str.), and the other comprising chemically and anatomically divergent taxa producing 2'-O-methylperlatolic acid, now accommodated in *Chicitaea* (Lumbsch et al. 2007; Guzow-Krzemińska et al. 2018; Ptach-Styn et al. 2024).

*Coniocarpon* DC. (Arthoniaceae Rchb.) was first described by De Candolle (De Lamarck and De Candolle 1805), who noted its thallus surface as bearing nodules of lentil-like granules covered with a friable colored powder and that the nodules reveal a raised or flattened surface when the powder falls off. *Coniocarpon cinnabarinum* DC. was later designated as the type species by Santesson (1952). Based on the phylogenetic analysis, *Coniocarpon* was confirmed as monophyletic by Frisch et al. (2014). Species of *Coniocarpon* are characterized by a smooth, immersed to erumpent thallus, sometimes delimited by a dark line. The ascomata vary from rounded to irregularly lirellate and contain orange, red or purple crystalline quinoid pigments, which dissolve in 10% potassium hydroxide to form a purple solution. The asci are of the *Arthonia*-type, typically containing eight ascospores which are hyaline, transversely septate and macrocephalic with large apical cells that turn brownish at maturity and develop granular epispore ornamentation (Cannon et al. 2020; Frisch et al. 2020). Currently, more than 20 species of *Coniocarpon* have been reported worldwide (Table 4), including three from China. (Wei 2017).

The genus *Synarthonia* Müll. Arg. was firstly described and circumscribed by Müller et al. (1891) based on the type species *Synarthonia bicolor*, collected in Costa Rica. For a long time, the genus remained unassigned to any family. Broeck et al. (2018) sequenced several specimens from tropical African *Synarthonia* species, confirming the monophyly of the genus for the first time and resolving its taxonomic placement within the Arthoniaceae. *Synarthonia* is morphologically characterized by white-pruinose ascomata, including orange pruinose or non-pruinose ascomata. The asci contain eight spores with K/I+ blue ring like structures in the tholus, although this feature is variable. The ascospores are transversely septate with an enlarged apical cell (Cannon et al. 2020). To date, about 25 species of *Synarthonia* have been reported worldwide (Table 5), but none have been reported from Guizhou, China.

During a recent field survey in Guizhou Province, southwestern China, two previously undescribed species were discovered. One belongs to the genus *Chicitaea*, representing the first record of this genus from China and the other belongs to *Coniocarpon*. Here, we describe two new species based on morphological, chemical and molecular data (ITS and mtSSU) and provide a brief checklist of related taxa.

## Material and methods

### Sample collection & morphological observations

The samples were collected from the Yueliangshan Nature Reserve in Congjiang county and the Chishui National Nature Reserve in Guizhou Provinces, China. All voucher specimens are preserved in the Lichen Herbarium Kunming Institute of Botany (KUN-L), Chinese Academy of Sciences, China. Anatomical features were examined using a stereomicroscope (OLYMPUS SZX16, JAPAN) equipped with a digital camera (AOR B32, CHINA) for image acquisition. Hand-cut longitudinal sections of apothecia were prepared using a razor blade and observed under a compound microscope (OLYMPUS BX53, JAPAN). The sections were mounted in distilled water and images were captured using a digital camera (OLYMPUS DP72, JAPAN). Potassium iodine solution (abbreviated IKI) was used to stain and examine the hymenium for detecting amyloid reactions. Ascospores and ascocarps dimensions were measured using ImageJ software (v. 1.50d) and photographic plates were prepared with Adobe Photoshop CC 2019 (Adobe, USA). Measurements are presented as (min–) (x̄ – SD) – (x̄ + SD) (–max), where ‘min’ and ‘max’ are the observed extreme values, x̄ is the arithmetic mean and SD is the standard deviation.

### Chemical component analysis

Color spot tests were performed on the thallus and medulla using 10% potassium hydroxide solution (KOH, abbreviation K), saturated sodium hypochlorite (NaClO, abbreviation C) and a saturated solution of *p*-phenylenediamine in 95% ethanol (P). The secondary metabolites were identified by thin-layer chromatography (TLC) using solvent system B’ (Hexane/Methyl tert-butyl ether/Formic acid = 140/72/18, *v*/*v*/*v*), and C (Formic acid/Acetic acid = 200/30, *v*/*v*). The analysis was performed using a standard sample applicator, with atranorin acid (Rf class 7) and norstictic acid (Rf class 4) as reference standards. *Lethariella cladonioides* (containing norstictic acid and atranorin) was set as the standard sample (Culberson 1972).

### DNA extraction, PCR amplification & sequencing

Genomic DNA was extracted directly from apothecia using a fungal genomic DNA extraction kit (Solarbio, China), following the manufacturer’s instructions. The primer pairs mrSSU1/mrSSU3R and ITS1/ITS4 were employed to amplify sequences of mitochondrial small subunit rRNA (mtSSU) and the internal transcribed spacer region of rDNA (ITS) (Vilgalys and Hester 1990; Kraichak et al. 2015). Polymerase Chain Reaction (PCR) was performed using a Mastercycler (Bio-RAD T-100) for 25-μL reactions with 12.5 μL of 2×Mix (Solarbio, China dNTPs Mix), 8 µL of double-distilled water (ddH_2_O), 1.0 µL for each 10 mM primer and 2.5 µL of DNA template. The PCR conditions were as follows: (1) for mtSSU, initial denaturation at 95 °C for 5 min, denaturation at 94 °C for 45 s, annealing at 50 °C for 60 s, elongation at 72 °C for 90 s, followed by 38 cycles, and a final extension at 72 °C for 10 min, then hold at 4 °C/+∞. (2) for ITS, the other steps are the same as (1) mtSSU, except for the annealing step, which is at 55 °C for 60 s (Kraichak et al. 2015). PCR products were detected by 1% agarose gel electrophoresis and sequenced by Beijing Tsingke Biotech Co., Ltd. (Chongqing, China).

### Phylogenetic analyses

**Figure 1.**
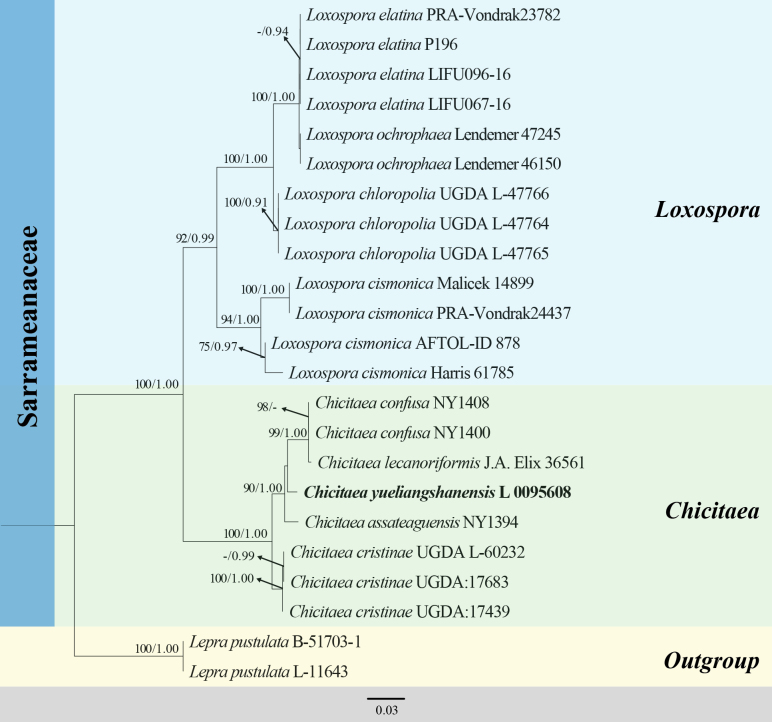
RAxML analysis of the Sarrameanaceae based on combined ITS and mtSSU sequence data. Bootstrap support values for maximum likelihood (ML ≥ 70%), and the Bayesian Posterior Probabilities (PP ≥ 0.90) are shown near the nodes as ML/PP. Hyphen (-) represents support values < 70% ML and < 0.90 PP. *Lepra pustulata* (B-51703-1) and *L. pustulata* (L-11643) are used as outgroup taxa. The newly generated sequences are shown in bold.

**Figure 2.**
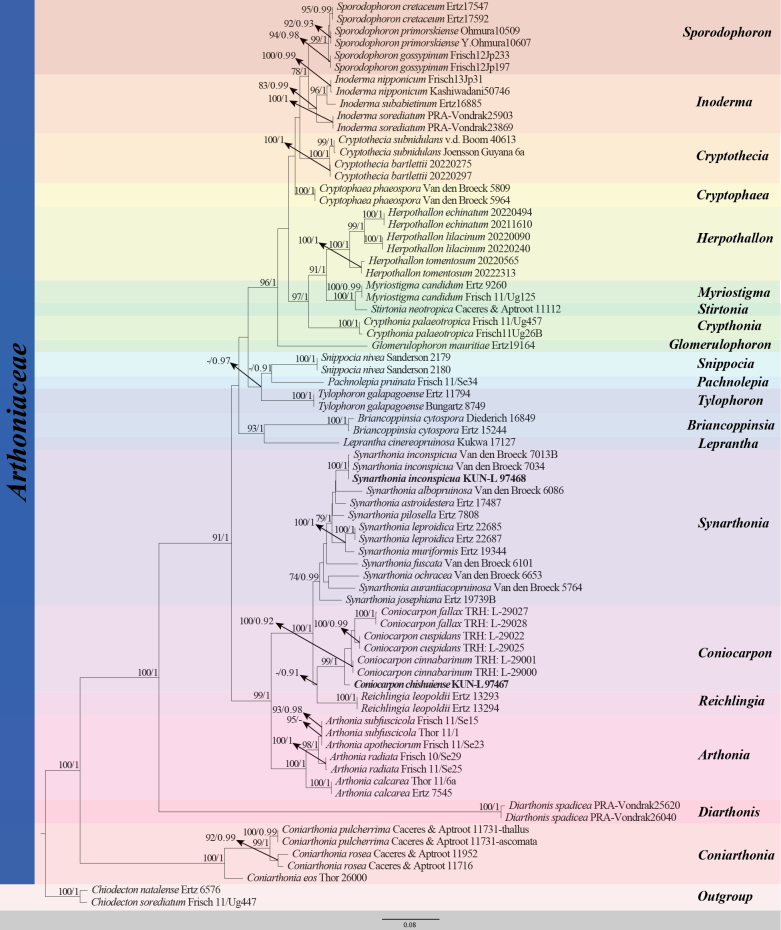
RAxML analysis of the Arthoniaceae based on mtSSU sequence data. Bootstrap support values for maximum likelihood (ML ≥ 70%), and the Bayesian Posterior Probabilities (PP ≥ 0.90) are shown near the nodes as ML/PP. Hyphen (-) represents support values < 70% ML or < 0.90 PP. *Chiodecton sorediatum* (Frisch 11/Ug447), *Chiodecton natalense* (Ertz 6576) are used as outgroup taxa. The newly generated sequences are shown in bold.

The sequencing chromatograms were analyzed using the BioEdit sequence alignment software (Version 7.0.9.0) to assess the quality of the sequence results. Forward and reverse sequences were assembled using ContigExpress software (Version 6.0.620.0). The preliminary taxonomic affiliation and the potential sample contamination were confirmed using BLASTn searches (https://blast.ncbi.nlm.nih.gov/Blast.cgi). Newly generated sequences were deposited in GenBank (Table 1 and Table 2). Additional sequences used for ingroup analysis were retrieved from the NCBI website (https://www.ncbi.nlm.nih.gov). *Lepra pustulata* was used as outgroup taxa in the phylogenetic analyses (Fig. 1). Two outgroup species were chosen based on Aptroot et al. (2024): *Chiodecton natalense* (Roccellaceae), *Chiodecton sorediatum* (Roccellaceae) (Fig. 2). Phylogenetic analyses were conducted using the OFPT program (Zeng et al. 2023) following its protocol: each gene region’s dataset was initially aligned using the ‘auto’ strategy (based on data size) in MAFFT (Katoh and Standley 2013) and subsequently trimmed using the ‘gappyout’ method (based on gap distribution) in TrimAl (Capella-Gutiérrez et al. 2009). The best-fit nucleotide substitution models for each dataset were selected based on the Bayesian Information Criterion (BIC) from 22 common DNA substitution models with rate heterogeneity using ModelFinder (Kalyaanamoorthy et al. 2017). All datasets were concatenated with partition information for subsequent phylogenetic analyses. The consensus tree was summarized based on the extended majority rule. Additionally, maximum likelihood (ML) analysis was conducted using RAxML-HPC2 on ACCESS (8.2.12) in CIPRES Science Gateway (https://www.phylo.org/portal2/login!input.action) with the GTRGAMMA model and a rapid bootstrap analysis of 1000 replicates (Miller et al. 2010; Stamatakis 2014). Bayesian inference was performed using MrBayes (Ronquist et al. 2012), with two parallel Metropolis-coupled Markov Chain Monte Carlo runs (one ‘cold’ chain and three heated chains), sampling trees every 1000 generations. The run was automatically terminated when the average standard deviation of split frequencies dropped below 0.01, and the resulting tree was summarized after discarding the first 25% samples as burn-in. The resulting trees were visualized in FigTree v1.4.4 and further edited in Adobe Illustrator CC 2019.

**Table 1.** Taxon, locality, voucher specimen/strain, and corresponding GenBank accession numbers for the phylogenetic analysis of Sarrameanaceae in this study. The newly generated sequences are shown in bold. The absence of GenBank accession numbers is shown by “NA”.

Taxon	Locality	Voucher/Strain	GenBank Accessions Number	
			ITS	mtSSU
*Chicitaea assateaguensis*	USA	NY1394	KF028593	KF028590
*C. confusa*	USA	NY1400	KF028594	KF028591
*C. confusa*	USA	NY1408	KF028595	KF028592
*C. cristinae*	Poland	UGDA L-60232	PP080087	PP080130
*C. cristinae*	Poland	UGDA:17683	MF804266	MF804268
*C. cristinae*	Poland	UGDA:17439	MF804265	MF804267
*C. lecanoriformis*	Australia	J.A. Elix 36561	NA	EF525279
***C. yueliangshanensis***	**China**	**L 0095608**	**PV351458**	**PV351459**
*Lepra pustulata*	USA	B-51703-1	NA	MF109166
*L. pustulata*	USA	L-11643	NA	MF109165
*Loxospora chloropolia*	Poland	UGDA L-47766	PP080096	PP080133
*L. chloropolia*	Poland	UGDA L-47765	PP080095	PP080132
*L. chloropolia*	Poland	UGDA L-47764	PP080094	PP080131
*L. cismonica*	Romania	Malicek 14899	NA	PP080135
*L. cismonica*	Canada	Harris 61785	PP080098	PP080134
*L. cismonica*	Czech Republic	PRA-Vondrak24437	OQ717930	OQ646299
*L. cismonica*	USA	AFTOL-ID 878	HQ650640	DQ986899
*L. elatina*	Switzerland	LIFU067-16	KX132976	NA
*L. elatina*	Switzerland	LIFU096-16	KX133004	NA
*L. elatina*	Austria	P196	KR017063	KR017350
*L. elatina*	Czech Republic	PRA-Vondrak23782	OQ717931	OQ646300
*L. ochrophaea*	USA	Lendemer 46150	PP080113	PP091207
*L. ochrophaea*	USA	Lendemer 47245	PP080112	PP080144

**Table 2.** Taxon, locality, voucher specimen/culture strain, and corresponding GenBank accession numbers for the phylogenetic analysis of Arthoniaceae. The newly generated sequences are shown in bold.

Taxon	Locality	Voucher/Strain	GenBank Accessions Number mtSSU
*Arthonia apotheciorum*	Sweden	Frisch 11/Se23	KJ850970
*A. calcarea*	France	Ertz 7545	EU704063
*A. calcarea*	Sweden	Thor 11/6a	KJ850974
*A. radiata*	Sweden	Frisch 11/Se25	KJ850969
*A. radiata*	Sweden	Frisch 10/Se29	KJ850968
*A. subfuscicola*	Sweden	Thor 11/1	KJ850971
*A. subfuscicola*	Sweden	Frisch 11/Se15	KJ850972
*Briancoppinsia cytospora*	Belgium	Ertz 15244	JF830772
*B. cytospora*	Luxembourg	Diederich 16849	JF830771
*Chiodecton natalense*	Zambia	Ertz 6576	EU704051
*C. sorediatum*	Uganda	Frisch 11/Ug447	KF707648
*Coniarthonia eos*	Japan	Thor 26000	KJ850987
*C. pulcherrima*	Brazil	Cáceres & Aptroot 11731-thallus	KP843610
*C. pulcherrima*	Brazil	Cáceres & Aptroot 11731-ascomata	KP843609
*C. rosea*	Brazil	Cáceres & Aptroot 11952	KP843608
*C. rosea*	Brazil	Cáceres & Aptroot 11716	KP843607
***Coniocarpon chishuiense***	**China**	**L 97467**	**PV990243**
*C. cinnabarinum*	Norway	TRH: L-29001	MN733981
*C. cinnabarinum*	Norway	TRH: L-29000	MN733980
*C. cuspidans*	Norway	TRH: L-29025	MN733976
*C. cuspidans*	Norway	TRH: L-29022	MN733974
*C. fallax*	Norway	TRH: L-29028	MN733965
*C. fallax*	Norway	TRH: L-29027	MN733964
*Crypthonia palaeotropica*	Uganda	Frisch11Ug26B	KP870145
*C. palaeotropica*	Uganda	Frisch 11/Ug457	KJ850961
*Cryptophaea phaeospora*	DRC	Van den Broeck 5809	KX077541
*C. phaeospora*	DRC	Van den Broeck 5964	KX077540
*Cryptothecia bartlettii*	China	20220297	PP051262
*C. bartlettii*	China	20220275	PP051261
*C. subnidulans*	Guyana	Joensson Guyana 6a	KJ850953
*C. subnidulans*	Réunion	v.d. Boom 40613	KJ850952
*Diarthonis spadicea*	Czech Republic	PRA-Vondrak25620	OQ646095
*D. spadicea*	Austria	PRA-Vondrak26040	OQ682860
*Glomerulophoron mauritiae*	Mauritius	Ertz19164	KP870153
*Herpothallon echinatum*	China	20211610	OQ676540
*H. echinatum*	China	20220494	OQ676537
*H. lilacinum*	China	20220090	OQ676532
*H. lilacinum*	China	20220240	OQ676529
*H. tomentosum*	China	20222313	OQ676539
*H. tomentosum*	China	20220565	OQ676538
*Inoderma nipponicum*	Japan	Kashiwadani50746	KP870149
*I. nipponicum*	Japan	Frisch13Jp31	KP870148
*I. sorediatum*	Czech Republic	PRA-Vondrak23869	OQ646251
*I. sorediatum*	Czech Republic	PRA-Vondrak25903	OQ646250
*I. subabietinum*	Azores	Ertz16885	KP870150
*Leprantha cinereopruinosa*	Poland	Kukwa 17127	MG207692
*Myriostigma candidum*	Gabon	Ertz 9260	EU704052
*M. candidum*	Uganda	Frisch 11/Ug125	KJ850959
*Pachnolepia pruinata*	Sweden	Frisch 11/Se34	KJ850967
*Reichlingia leopoldii*	Belgium	Ertz 13294	JF830774
*R. leopoldii*	Belgium	Ertz 13293	JF830773
*Snippocia nivea*	England	Sanderson 2180	MG207694
*S. nivea*	England	Sanderson 2179	MG207693
*Sporodophoron cretaceum*	France	Ertz17592	KP870152
*S. cretaceum*	France	Ertz17547	KP870151
*S. gossypinum*	Japan	Frisch12Jp197	KP870156
*S. gossypinum*	Japan	Frisch12Jp233	KP870155
*S. primorskiense*	Japan	Ohmura10607	LC086299
*S. primorskiense*	Russia	Ohmura10509	KP870157
*Stirtonia neotropica*	Brazil	Cáceres & Aptroot 11112	KP843611
*Synarthonia albopruinosa*	DRC	Van den Broeck 6086	MH251873
*S. astroidestera*	France	Ertz 17487	MT141113
*S. aurantiacopruinosa*	DRC	Van den Broeck 5764	MH251874
*S. fuscata*	DRC	Van den Broeck 6101	MH251875
*S. inconspicua*	Tanzania	Van den Broeck 7034	MH251882
*S. inconspicua*	Tanzania	Van den Broeck 7013B	MH251881
***S. inconspicua***	**China**	**L 97468**	**PV990244**
*S. josephiana*	Madagascar	Ertz 19739B	MH251876
*S. leproidica*	Luxembourg	Ertz 22687	MT141117
*S. leproidica*	Luxembourg	Ertz 22685	MT141115
*S. muriformis*	Madagascar	Ertz 19344	MH251877
*S. ochracea*	France	Van den Broeck 6653	MH251884
*S. pilosella*	Rwanda	Ertz 7808	MH251883
*Tylophoron galapagoense*	Galapagos Islands	Ertz 11794	JF830777
*T. galapagoense*	Galapagos Islands	Bungartz 8749	JF830776

## Result

### Phylogenetic analyses

The final dataset for Sarrameanaceae comprised 2 genera 10 taxa 23 strains/vouchers with 1118 aligned characters, including gaps (ITS 507 bp, mtSSU 611 bp). The RAxML tree was constructed with a final ML optimization likelihood value of -3361.20982. The parameters for the GTR+I+G model of combined ITS and mtSSU were as follows: estimated base frequencies: A = 0.29, C = 0.20, G = 0.22, T = 0.28, substitution rate AC = 1.44, AG = 3.40, AT = 1.62, CG = 1.15, CT = 7.07 and GT = 1.00. Bayesian posterior probabilities from MCMC analysis showed a final average standard deviation of split frequencies < 0.01 at 61000. The topologies from both ML and Bayesian analyses were compared manually and found to be largely congruent (Fig. 1). In the phylogenetic tree, the new species is recovered in a well-supported singleton with the four known species of the genus *Chicitaea*.

The complete dataset for *Coniocarpon* and *Synarthonia* (Arthoniaceae) comprised 46 taxa 75 strains/vouchers, including gaps (mtSSU 820 bp). The RAxML tree was constructed with a final ML optimization likelihood value of -8923.924427. The parameters for the GTR+I+G model of mtSSU were as follows: estimated base frequencies: A = 0.32, C = 0.15, G = 0.21, T = 0.31, substitution rate AC = 1.20, AG = 5.73, AT = 2.83, CG = 0.62, CT = 6.70 and GT = 1.00. Bayesian posterior probabilities from MCMC analysis showed a final average standard deviation of split frequencies < 0.01 at 276000 generations. The topologies from both ML and Bayesian analyses were compared manually and found to be largely congruent (Fig. 2). Based on the phylogenetic tree, the new species, *Coniocarpon chishuiense* is basal in the *Coniocarpon*-clade that it is related to *C. cinnabarinum* (TRH: L-29001 and TRH: L-29000), *Coniocarpon cuspidans* (Nyl.) Moen, Frisch & Grube (TRH: L-29022 and TRH: L-29025) and *Coniocarpon fallax* (Ach.) Grube (TRH: L-29028 and TRH: L-29027). The base divergence of *Coniocarpon chishuiense* to *C. cinnabarinum* is 11/747 bp, to *C. cuspidans* 15/747 bp and to *C. fallax* 27/747 bp. The new record *Synarthonia inconspicua* (L 97468) was recovered in a fully supported clade with *S. inconspicua* (Van den Broeck 7034 and Van den Broeck 7013B).

### Chemical analysis

TLC analysis of *Chicitaea yueliangshanensis* revealed three well-separated spots following development with solvent system B’. After spraying with 10% H_2_SO_4_ and heating until colors developed, the color of the spots are yellow under UV_365_ and sunlight. Based on the documented research of this genus (Culberson and Kristinsson 1970), this species contains perlatolic acid, 2’-O-methylperlatolic acid and an unknown compound.

Lichen substances in *Coniocarpon chishuiense* were detected by solvent systems C. After spraying with 10% H_2_SO_4_ and heating, distinct pink coloration was observed under both UV_365_ and sunlight which indicated that the species contains psoromic acid and an unidentified lichen substance (Culberson and Kristinsson 1970).

### Taxonomy

#### Chicitaea yueliangshanensis

Taxon classificationFungiSarrameanalesCorylaceae

Wei Wu & Shao-Bin Fu sp. nov.

CF823572-77BA-5282-B1E2-CA897FF55DF4

Index Fungorum: IF903587

Facesoffungi: FoF17619

Fig. 3

##### Etymology.

The specific epithet yueliangshanensis refers to the Yueliangshan Nature Reserve, where the holotype was collected.

**Figure 3.**
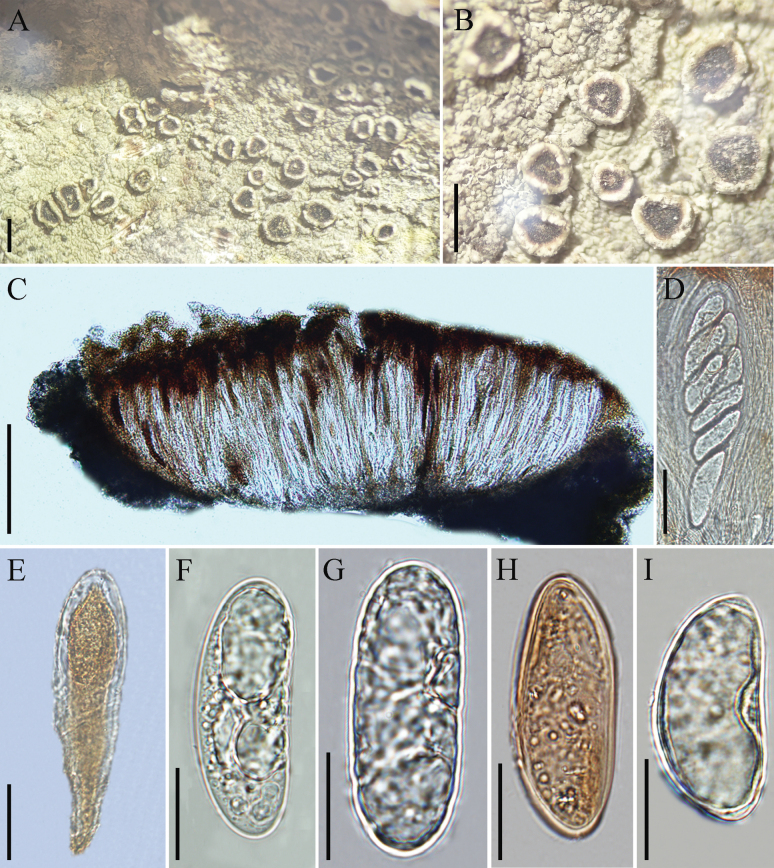
*Chicitaea yueliangshanensis* (KUN-L 0095608, holotype). **A, B**. Thallus with ascomata; **C**. Cross section of apothecium; **D, E**. Ascus; **F, G**. Ascospores (in water); **H**. Aging of ascospores; **I**. Ascospore (in IKI). Scale bars: 1 mm (**A, B**); 200 µm (**C**); 50 µm (**D, E**); 20 µm (**F–I**).

##### Holotype.

KUN-L 0095608

##### Description.

***Sexual morph*: *Thallus*** corticolous, crustose, thin, tightly attached to the substratum, pale gray-green to olive-gray, rough, dull, lacking isidia and soredia, prothallus absent. ***Apothecia*** lecanorine, prominent, numerous, sessile, concave, measuring 0.3–1.2 mm diam. ***Disc*** black, epruinose, ± flat. Proper margin conspicuous. ***Exciple*** uncarbonized. ***Epihymenium*** brown, 15–25 μm tall. ***Hymenium*** hyaline to reddish brown, not inspersed, 270–400 μm tall, I+ blue. ***Paraphyses*** simple, unbranched, with colorless apices. ***Asci*** 8-spored, hyaline, claviform to obovate, 175–212 × 45–65 μm. ***Ascospores*** broadly ellipsoid, straight or slightly bent (kidney shaped), (40.0–)46.5–57.5(–62.0) × (16.0–)17.5–22.0(–26.0) μm (x̄ = 52 × 20 μm, n = 35), I–, with a single thin wall, c.1 μm thick. ***Asexual morph***: not observed.

##### Chemistry.

Thallus K–, C–, KC–, P–, UV–. TLC: Perlatolic acid, 2’-O-methylperlatolic acid and an unknown lichen substance.

##### Material examined.

China • Guizhou Province, Congjiang County, Yueliangshan Nature Reserve, 25°24'37"N, 108°34'47"E, elev. 959 m, 23 October 2023, Bo Liu and Ze Yang, Coll. No. Y337 (KUN-L 0095608, holotype).

##### Notes.

Phylogenetic analysis based on combined ITS and mtSSU sequence place *Chicitaea yueliangshanensis* in a sister clade with *C. assateaguensis*, although with low support. The nucleotide comparison revealed a difference of 3.95% (19/486 bp) for ITS and 0.98% (6/611 bp) for mtSSU. Morphologically, *C. assateaguensis* differs by possessing soredia and lacking apothecia, whereas the new species lacks soredia and has apothecia. Chemically, *C. assateaguensis* contains only 2’-O-methylperlatolic acid, while the new species additionally contains perlatolic acid and an unknown lichen substance (Lendemer 2013).

Among the four species of *Chicitaea*, *C. lecanoriformis* is the only taxon with apothecia (Table 2). However, it differs from the new species by producing pycnidia (absent in the new species) and slight genetic divergence in the mtSSU region (0.81%; 5/611 bp). According to lichen substances, both species contain 2’-O-methylperlatolic acid and perlatolic acid, whereas the new species contains an unknown lichen substance (Lumbsch et al. 2007). Different from *C. confusa*, which has granulose isidia, the new species lacks this feature. Nucleotide comparisons revealed differences of 3.49% (17/486 bp) in the ITS region and 0.65% (6/611 bp) in the mtSSU region between the two species. The new species contains perlatolic acid, 2’-O-methylperlatolic acid and an unknown lichen substance, in contrast to *C. confusa*, which produces only 2’-O-methylperlatolic acid (Lendemer 2013). These combined morphological, chemical, and molecular differences support the recognition of *Chicitaea yueliangshanensis* as a distinct species.

#### Coniocarpon chishuiense

Taxon classificationFungiArthonialesArthoniaceae

Lin-Zhi He & Shao-Bin Fu sp. nov.

CE920A83-D7A5-55DE-BE86-A52CB023290A

Index Fungorum: IF904119

Facesoffungi: FoF18009

Fig. 4

##### Etymology.

The specific epithet chishuiense refers to the location (Chishui City) where the holotype was collected.

**Figure 4.**
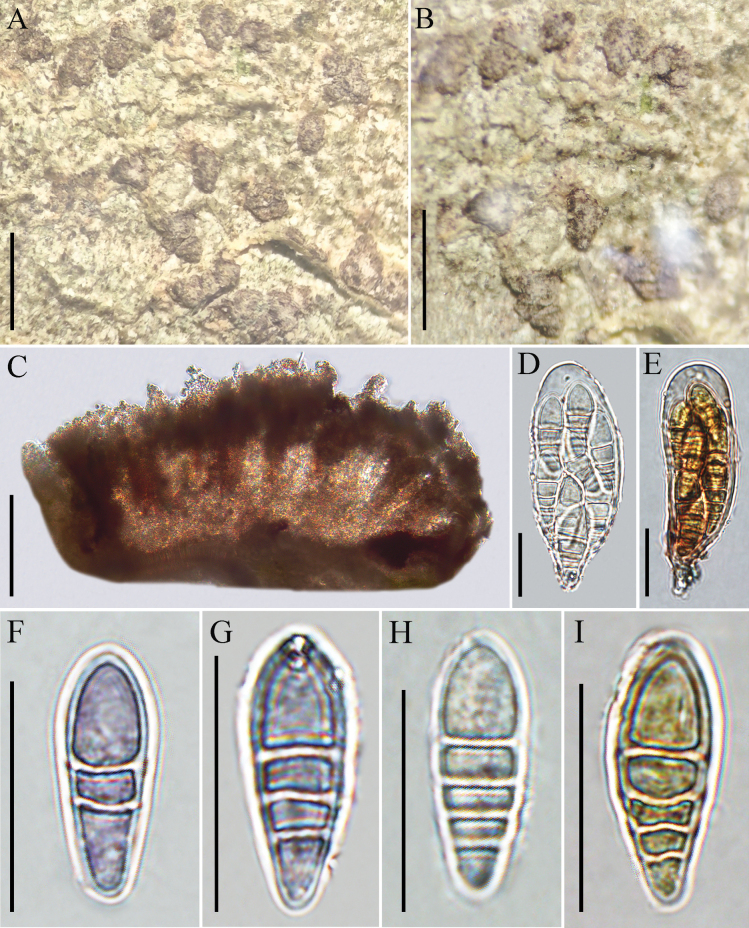
*Coniocarpon chishuiense* (KUN-L 97467, holotype). **A, B**. Thallus with ascomata; **C**. Cross section of apothecium; **D**. Ascus (in water); **E**. Ascus (in IKI); **F, G**. Ascospores (in water); **H**. Ascospores (in KOH); **I**. Ascospores (in IKI). Scale bars: 1 mm (**A, B**); 50 µm (**C**); 20 µm (**D–I**).

##### Holotype.

KUN-L 97467

##### GenBank.

PV990242 (ITS, KUN-L 97467); PV990243 (mtSSU, KUN-L 97467)

##### Description.

***Sexual morph*: *Thallus*** crustose, thin, olive, rough, dull, with obvious edge. ***Apothecia*** irregularly rounded to elliptical, numerous, convex, sessile, with steeply dipping, emergent from thallus, 0.3–0.5 mm × 0.2–0.3 mm, solitary or forming loose to dense. ***Disc*** sepia, epruinose, flat to weakly convex. ***Exciple*** brown, uncarbonized. ***Epihymenium*** brown, 28–39 μm tall, I+ blue, K–. ***Hymenium*** is hyaline to reddish brown, not inspersed, 104–165 μm tall, I+ blue then turning red, K–. ***Hypothecium*** brown, 31–48 μm tall, I+ blue, K–. ***Paraphyses*** simple, unbranched, with colorless apices. ***Asci*** 8-spored, hyaline, long obpyriform to clavate, (46.0–)54.0–70.0(–74.0) × (23.5–)23.5–27.5(–30.0) μm, I–, K–. ***Ascospores*** obovate, (2–)3–5(–5)-septate, showing purple (in water) and hyaline (in KOH), (20.5–)21.5–26.5(–29.0) × (6.5–)7.5–9.0(–9.5) μm (x̄ = 24 × 8 μm, n = 20), I–, K–. ***Asexual morph***: not observed.

##### Chemistry.

Thallus K+ blackish purple, C–, KC+ purple, P–, UV–. TLC: psoromic acid and an unknown lichen substance.

##### Material examined.

China • Guizhou Province, Chishui City, Chishui National Nature Reserve, 28°21'49"N, 105°59'51"E, 1125 m elev., on bark, 12 July 2024, ShaoBin Fu, WeiWei Zheng, Coll. No. CS78 (holotype, KUN-L 97467).

##### Notes.

The phylogenetic analysis of mtSSU sequence data reveals that *Coniocarpon chishuiense* is closely related to *C. cinnabarinum*, *C. cuspidans* in and *C. fallax*. The nucleotide comparison differences between *C. chishuiense* and *C. cinnabarinum*, showed differences of 1.47% (11/747 bp) for mtSSU. Morphologically, the new species differs by epruinose disc (*vs*. reddish or white pruina in *C. cinnabarinum*). In addition, the asexual moprh (pycnidia and conidia) is absent in new species but present in *C. cinnabarinum*. Chemically, *C. chishuiense* contains both psoromic acid and an unidentified lichen substance, while *C. cinnabarinum* contains only psoromic acid (Nash et al. 2004; Frisch et al. 2020).

There were 2.00% differences (15/747 bp) in sequences of mtSSU between *Coniocarpon chishuiense* and *C. cuspidans* in. Additional differences include the larger ascospores in the new species (21.5–26.5 × 7.5–9.0 μm *vs*. 16–18 × 7–8 µm in *C. cuspidans*) and the presence of psoromic acid and an unknown lichen substance in *C. chishuiense* but not in *C. cuspidans* (Frisch et al. 2020). *C. chishuiense* can be distinguished from *C. fallax* by its epruinose disc (vs. orange-red pruina in *C. fallax*) and larger ascospores (21.5–26.5 µm *vs*. 17–20 μm). Furthermore, *C. chishuiense* contains psoromic acid and an unknown lichen substance, both absent in *C. fallax*. Nucleotide comparison is also considerable, with differences of 3.61% (27/747 bp) in mtSSU (Frisch et al. 2020).

In addition to the above three species, the new species, *Coniocarpon chishuiense*, shares similar morphology with *Coniocarpon carneoumbrinum* (Zahlbruckner) Van den Broeck & Ertz, *Coniocarpon rubrocinctum* (G. Merr. ex Grube & Lendemer) Perlmutter, R. Miranda & Bungartz and *Coniocarpon tuckermanianum* (Willey) Van den Broeck & Ertz in size of ascospores (Table 4). However, all three of these species have a pruinose disc, whereas our species lacks pruina. A distinct contrast is observed in thallus coloration among the three species *C. carneoumbrinum*, *C. tuckermanianum* and *C. chishuiense*: gray *vs*. white *vs*. olive. Besides, *Coniocarpon rubrocinctum* contain only psoromic acid, while *C. chishuiense* contains an unknown lichen substance except for psoromic acid. Based on the descriptions of thallus and apothecial color, the new species is distinct from all known species.

#### Synarthonia inconspicua

Taxon classificationFungiArthonialesArthoniaceae

(Stirton) Van den Broeck & Ertz

1D794733-CE4B-5CBE-BD70-F27DA2C995AC

Fig. 5

##### Description.

***Sexual morph*: *Thallus*** crustose, thin, pale green, smooth, glossy, with obvious edge. ***Apothecia*** round to irregular shape, intensive, numerous, weakly concave, sessile, disk white, often forming irregular clusters of 0.4–0.7 × 0.2–0.5 mm. ***Disc*** brownish, with white pruina, flat to concave. ***Exciple*** uncarbonized. ***Epihymenium*** dark brown, 20–34 μm tall. ***Hymenium*** hyaline, 59–85 μm tall, not inspersed, I+ reddish, K–, K/I+ blue. ***Hypothecium*** brown, 11–24 μm tall. ***Paraphyses*** simple, unbranched, with colorless apices. ***Asci*** 8-spored, hyaline, clavate, obovoid to ellipsoid, or (sub-) globose, with top ring structure, (37.0**–**)40.5**–**51.5(**–**52.50) × (20.5–)21.5–25.5(–26.0) μm (n = 10), I+ reddish, K–, K/I–. ***Ascospores*** obovate, oblong-ovoid, 3-septate, (16.0–)17.5–20.0(–21.0) × (5.5–)6.0–6.5(–7.0) μm (n = 20), I–, K–, K/I–. ***Asexual morph***: not observed.

**Figure 5.**
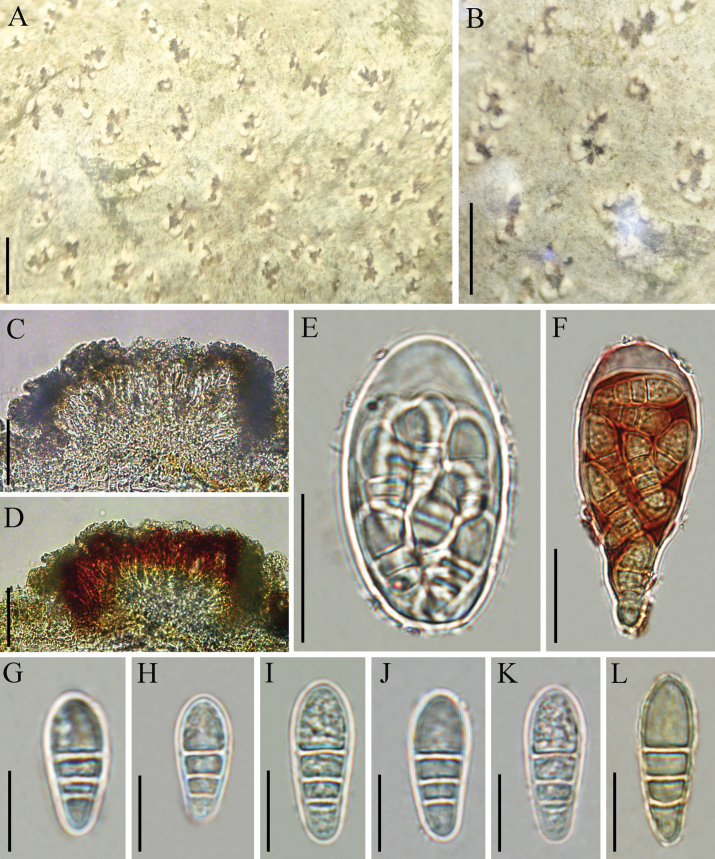
*Synarthonia inconspicua* (KUN-L 97468). **A, B**. Thallus with ascomata; **C**. Hymenium (in water); **D**. Hymenium (in IKI); **E**. Ascus (in water); **F**. Ascus (in IKI); **G–K**. Ascospores (in water); **L**. Ascospores (in IKI). Scale bars: 1 mm (**A, B**); 100 µm (**C, D**); 20 µm (**E, F**), 10 µm (**G–L**).

##### Chemistry.

Thallus K–, C–, KC–, Pd–, UV–. TLC: No lichen products detected.

##### Material examined.

China • Guizhou Province, Chishui City, Chishui Alsophila National Nature Reserve, 28°21'51"N, 105°59'55"E, 525 m elev., on bark, 12 July 2024, ShaoBin Fu, WeiWei Zheng, Coll. No. CS80 (KUN-L 97468).

##### Notes.

In the phylogenetic analyses, our collection forms a well-supported clade (100% bootstrap support) with *Synarthonia inconspicua* (Van den Broeck 7034, Van den Broeck 7013B). Excluding gaps, no nucleotide difference was detected in the mtSSU (770 bp) region between our collection and reference specimens. Morphologically, this specimen agrees well with the description of *S. inconspicua* by Broeck et al. (2018), particularly in the presence of white pruina on the disc and in the size of ascospores (17.5–20.0 × 6.0–6.5 μm vs. 16.5–21.3 × 6.2–7.7 μm) (Broeck et al. 2018). Therefore, we identify this collection as *Synarthonia inconspicua*. This represents the first record of this species in Guizhou, China.

## Discussion

Species identification in lichenized fungi is a complex taxonomic undertaking that requires a multifaceted approach. In this study, the newly collected species were identified by an integrated method that includes phylogenetic analyses, detailed morphological characteristics and chemical profiling. Additionally, we conducted a comprehensive literature review and compiled the diagnostic characteristics of *Chicitaea* (Table 3), *Coniocarpon* (Table 4) and *Synarthonia* (Table 5).

**Table 3.** Morphological Characteristics list of *Chicitaea* species.

Species name		Soredia	Ascomata	Hymenium	Pycnidia	Chemistry	References	Notes
1	*Chicitaea assateaguensis* Lendemer	soralia erumpent, circular, without a discrete rim; soredia coarse, 30–70 μm in diameter	apothecia and pycnidia not observed. Photobiont coccoid, cells 5–10 μm in diameter			2’-0-methylperlatolic acid. Spot tests: K–, C–, KC–, P–, UV+ blue-white	Lendemer (2013)	= *~~~PROTECTED_TN_221~~~ assateaguensis*
2	*Chicitaea confusa* Lendemer	100–200 μm tall and ~50 μm wide	apothecia and pycnidia not observed. Photobiont coccoid, cells 4–7 μm in diameter			2’-0-methylperlatolic acid. Spot tests: K–, C–, KC–, P–, UV+ blue-white	Lendemer (2013)	= *~~~PROTECTED_TN_223~~~ confusa*
3	*Chicitaea cristinae* Guzow-Krzemińska, Łubek, Kubiak & Kukwa	soredia white to greenish gray, sometimes pale brownish white, 35–75 μm in diam., simple or more often in consoredia	ascomata and conidiomata unknown. Photobiont trebouxioid, cells 7–10 μm in diam			2’-O-methylperlatolic acid. Spot tests: K–, C–, P–, UV–, medulla K–, C–, P–; UV+ whitish	Guzow-Krzemińska et al. (2018)	= *~~~PROTECTED_TN_225~~~ cristinae*
4	*Chicitaea lecanoriformis* Lumbsch, A. W. Archer & Elix	–	apothecia lecanorine, 0.8–1.5 mm diam., disc dark reddish brown to black, epruinose, ± flat	hymenium colorless, 350–500 μm tall, inspersed with infrequent oil droplets to 12 μm diam., K–, I–, KI– or slightly greenish blue; asci 6–8-spored, ascospores I–, KI+ slightly blue-green, 50–65 × 8–22 μm	pycnidia visible as minute black dots, c. 0.05 mm wide. Conidia bacilliform, 3–4.5 × 0.5–0.7 μm	2’-O-methylperlatolic acid (major) and perlatolic acid (minor or trace). Cortex K–, C–, KC–, P–, UV–, medulla K–, C–, KC–, P–, UV+ white	Lumbsch et al. (2007)	= *~~~PROTECTED_TN_227~~~ lecanoriformi*

**Table 4.** Morphological Characteristics list of *Coniocarpon* species.

Species name		Thallus	Ascomata	Ascospores num./size	Pycnidia	Chemistry	References	Notes
1	*Coniocarpon affine* A. Massal.	slightly elevated, almost smooth, violet-lilac in color	punctiform (dot-like), black, somewhat rounded-irregular	8-spored, 12.2–18.5 × 5.66 µm	–	–	Massalongo (1853)	= *~~~PROTECTED_TN_230~~~ affinis*
2	*Coniocarpon caribaeum* Fée	somewhat powdery; grayish to brown, well-defined, sometimes spreading	–	–	–	–	Fée (1825)	–
3	*Coniocarpon carneoumbrinum* (Zahlbruckner) Van den Broeck & Ertz	gray	rounded, convex, sessile; disc heavily white to pink pruinose	24.0–28.5 × 8.0–10.0 µm	–	–	Broeck et al. (2018)	= *~~~PROTECTED_TN_233~~~ carneoumbrina*
4	*Coniocarpon cascarillae* Fée	(crust) thin, very white, smooth, spreading	–	–	–	–	Fée (1825)	–
5	*Coniocarpon cinnabarinum* DC.	pale olive gray to brown, weakly glossy to matt, smooth, endophloeodal to partly epiphloeodal, continuous	irregularly rounded to elliptical to weakly lobed, rarely distinctly lirellate, 0.1–0.4 × 0.1–0.3 mm, old ascomata sometimes epruinose; disc dark purple black, flat to weakly convex, matt to weakly glossy, white pruinose, a layer of orange–red pruina sometimes present above the white pruina	8-spored, (19–)23–28(–30) × (8–)10–11(–12) µm	pycnidia very rare, ± round, 110–130 µm in diam.; conidia hyaline, non-septate, bacilliform, 4–6 × 1–1.5 µm	psoromic acid	Nash et al. (2004); Frisch et al. (2020)	= *~~~PROTECTED_TN_236~~~* = *~~~PROTECTED_TN_237~~~ gregarium*
6	*Coniocarpon coralloideum* Kalb & J.E. Hern.	corticolous, delimited by a fluffy reddish-brown line, off-white or pale gray, surface smooth with purple-red coralloid outgrowths	apothecia rare, 0.2–0.3 mm diam., flat to slightly concave, rounded, ± polygonal or linear; disc purple-brown, densely white pruinose	8-spored, 18–20 × 7–8 μm	–	two unknown violet to red pigments	Kalb et al. (2012)	–
7	*Coniocarpon cuspidans* (Nyl.) Moen, Frisch & Grube	pale brown to pale fawn to off white, matt to weakly glossy, smooth, endophloeodal to partially epiphloeodal, continuous	weakly elongate to irregularly lirellate, with steep flanks, emergent from thallus, 0.2–0.6 × 0.1–0.2 mm; disc black to dark purple black, flat to weakly convex, weakly glossy to matt, epruinose, rarely with patches of a thin white pruina	8-spored, (15–)16–18(–20) × (6–)7–8(–9) µm		red and purple crystals dissolve in K with purplish solution	Frisch et al. (2020)	= *Arthonia cinnabarina* f. cuspidans
8	*Coniocarpon fallax* (Ach.) Grube	pale fawn to gray brown, weakly glossy to matt, smooth, endophloeodal to partly epiphloedal, continuous	weakly elongate to irregularly lirellate, with steep flanks, 0.2–0.4 mm × 0.1–0.2 mm; disc black to dark purple black, flat to weakly convex, weakly glossy to matt, epruinose or with a thin layer of white pruina	8-spored, (15–)17–20(–22) × (6–)7–9(–10) µm	–	orange, red and purple crystals dissolve in K with purplish solution	Frisch et al. (2020)	= *Coniocarpon elegans* = *Spiloma fallax*
9	*Coniocarpon ferrugineum* Delise	thin, somewhat nail-like	scattered or confluent, minute, irregularly discoid to oblong-deformed, rough, sorediate	–	–	–	De Candolle (1828)	–
10	*Coniocarpon foliicola* Aptroot	crustose, continuous, not corticate, dull, pale ochraceous, covering areas of up to 1.5 cm, under 0.1 mm thick	solitary, superficial on the thallus, round to irregular oval, 0.2–0.5 mm diam.; disc pink, margin steep but internally not differentated	8-spored, 11.5–13 × 5–6 μm	–	no TLC performed (material too small). Spot tests: thallus C–, K–, KC–, P–	Aptroot et al. (2022)	–
11	*Coniocarpon gracile* (Eschw.) A. Massal.	laminose, whitish	very minute, crowded, linear, flexuose, bifid or stellate-branched, more rarely punctiform, wine-red, ultimately becoming prominent	–	–	–	Eschweiler et al. (1829)	= *Ustalia gracilis*
12	*Coniocarpon nigrum* DC.	milk-white, spreading, slightly cracked	powder falls away a small, flattened, and inconspicuous disc is revealed	–	–	–	De Lamarck and De Candolle (1805)	–
13	*Coniocarpon olivaceum* DC.	whitish and scarcely visible	rounded, slightly convex, covered with a copious granular powder	–	–	–	De Lamarck and De Candolle (1805)	–
14	*Coniocarpon piccolioides* Aptroot & M. Cáceres	crustose	round to irregularly angular in outline, 0.2–0.5 mm diam.; disc convex, black but with a thick layer of golden pruina	8-spored, 11–12 × 4–4.5 μm	pycnidia black, at the tips of micro-squamules	an unidentified anthraquinone. Spot tests: thallus and disc K+ blood red, P–	Aptroot and Cáceres (2018)	–
15	*Coniocarpon radiatum* A. Massal.	powdery, whitish to pinkish, well-delimited	star-shaped, slightly swollen, dark brownish-black with a reddish tinge	12.2 × 2.4μm	–	–	Massalongo (1852)	–
16	*Coniocarpon rubrocinctum* (G. Merr. ex Grube & Lendemer) Perlmutter, R. Miranda & Bungartz	endoperidermal to epiperidermal, thin, effuse	sessile, elongate to furcate, lirellate with ± straight branches and acute tips, (0.4–) 1.0 (–1.6) × (0.2–) 0.3 (–0.5) mm; disc flat, brown, often covered with whitish pruina	8-spored, (15.5–) 22.0 (–26.0) × (4.0–) 5.5 (–8.0) µm	pycnidia frequent, ± globose, partly immersed, (52–) 80 (–140) µm; conidia bacilliform, mostly straight, hyaline, (4–) 6.4 (–9) × 1 µm	psoromic acid. Spot tests: thallus UV–, K–, C–, KC–, PD+ golden yellow	Perlmutter et al. (2023)	= *~~~PROTECTED_TN_251~~~*
17	*Coniocarpon sphaerale* Duby	absent (non-lichenized)	subglobose, rough, black, opaque, minute	–	–	–	De Candolle (1828)	–
18	*Coniocarpon subrotundum* (Fée) R. Meissn.	membranous, thin, white to grayish, spreading, sparsely marked with black lines	round, black, regular, slightly dish-shaped; disc marginless	–	–	–	Massalongo (1852)	= *~~~PROTECTED_TN_254~~~ subrotunda*
19	*Coniocarpon torulosum* (Fée) Fée	membranous-smooth, whitish-gray, bordered by a black line	black, irregular, elongated-torulose (beaded), blunt-tipped	–	–	–	Fée (1825)	= *~~~PROTECTED_TN_256~~~ torulosa*
20	*Coniocarpon tricolor* (Ach.) Duby	subtartareous (slightly rough), cracked, powdery, white	subrotund, convex, aggregated, confluent, rufous, when worn becoming ferrugineous-flavescent	–	–	–	De Candolle (1828)	= *Spiloma tricolor*
21	*Coniocarpon tuckermanianum* (Willey) Van den Broeck & Ertz	white, continuous, numerous	semi-immersed, reddish brown to black; disc occasionally with some white pruina	17.0–23.5 × 6.0–8.0 µm	–	–	Broeck et al. (2018)	–
22	*Coniocarpon vulgare* Rabenh	irregularly spreading, very thin, sometimes absent, often tomentose, continuous, whitish, finally becoming farinose and thalline	scale-like, brown, margins nearly black, later often black	–	–	–	Rabenhorst (1845)	–
23	*Coniocarpon xanthostigma* (Ach.) Duby	grayish-black	somewhat plane, punctiform, powdery, yellowish-green	–	–	–	De Candolle (1828)	*= Spiloma xanthostigma* = *Coniocarpon xanthostigmum*

**Table 5.** Morphological Characteristics list of *Synarthonia* species.

**Species name**		**Thallus**	**Ascomata**	**Ascospore num./size**	**Pycnidia**	**Chemistry**	**References**	**Notes**
1	*Synarthonia albopruinosa* Van den Broeck & Ertz	10–20 µm thick, green, smooth to farinose	solitary, 0.1–0.4 × 0.1–0.2 mm, often more or less grouped in elongated clusters; disc heavily white pruinose, especially at the margins	12.5–17.5 × 5.0–6.5 µm	–	an unknown substance. Spot tests: thallus K–, C–, KC–, PD+ orange (pruina and thallus)	Broeck et al. (2018)	–
2	*Synarthonia astroidestera* (Nyl.) Ertz & Van den Broeck	immersed, usually delimited by a brown line, cream-white to cream-yellow	linear (to 1.2 mm long), irregularly branched or stellate (to 1.2 mm diam.); disc usually thickly white-pruinose	(17–) 18–24 (–27) × 6–7 μm	–	spot tests: thallus C–, K–, KC–, Pd–, UV+ orange; pruinose apothecia UV+ bright orange-yellow (lichexanthone)	Broeck et al. (2018)	= *~~~PROTECTED_TN_265~~~ astroidestera*
3	*Synarthonia aurantiacopruinosa* Van den Broeck & Ertz	5–20 µm thick, inconspicuous; thallus hyphae not observed	solitary, 0.2–0.3 × 0.1–0.3 mm, rounded to elongate; disc black, matt, orange pruinose, rough, flat to concave	15.0–19.0 × 5.5–6.5 µm	–	without lichen substances. Spot tests: thallus K–, KC–, C–, PD–, UV–; pruina K+ purplish	Broeck et al. (2018)	–
4	*Synarthonia bicolor* Müll. Arg.	corticolous, crustose, white to whitish gray, smooth, 50–80 µm thick	solitary when young, with a well-developed white thalline margin; disc pale brownish to brownish, epruinose or rarely white pruinose	8-spored, (17.2–)20.0–24.0(–26.8) × (5.0–)7.0–8.5(–9.2) µm	–	lichexanthone present. Spot tests: thallus K–, C–, P–, UV+ yellow	Joseph and Sinha (2015)	–
5	*Synarthonia borbonica* (Ertz, Elix & Grube) Van den Broeck & Ertz	thin, continuous, smooth, white, matt, c. 20–80 µm thick	numerous, entirely covered by an orange pruina, 0.2–1(–1.5) × 0.12–0.2 mm; disc covered by a thin layer of orange pruina	8-spored (14–)15–18(–20) × (5–)6–7(–7.5) µm	–	ascomata contain parietin (major), xanthorin (minor), psoromic acid (major); thallus contains psoromic acid (major), 2’-O-demethylpsoromic acid (minor). Spot tests: thallus K–, C–, KC–, PD+ yellow, UV+ orange; apothecia K+ violet-red, C–, P–, UV+ orange intensifying	Ertz et al. (2010)	= *~~~PROTECTED_TN_269~~~*
6	*Synarthonia ferruginea* (Vain.) Van den Broeck & Ertz	white, smooth, ± continuous	solitary, 0.6–1.9 × 0.4–0.7 mm, immersed, erumpent, closely spaced; disc black with marginal orange pruina, orange brown when wet, flat	19.5–27.0 × 7.0–9.5 µm	–	without lichen substances. Spot tests: thallus UV+ orange	Broeck et al. (2018)	= *~~~PROTECTED_TN_271~~~ ferruginea*
7	*Synarthonia fuscata* Van den Broeck & Ertz	5–60 µm thick, slightly greenish with white striae or spots, smooth, continuous	solitary, not in clusters, 0.2–0.5 × 0.1–0.3 mm, elongate to irregularly lobed, numerous; disc light brown, epruinose, flat	13.5–17.0 × 4.5–6.5 µm	–	without lichen substances. Spot tests: thallus K–, KC–, C–, PD–, UV–	Broeck et al. (2018)	–
8	*Synarthonia hodgesii* (Lendemer & R.C. Harris) Van den Broeck & Ertz	elongate and irregularly shaped, 0.25–0.5 3 0.1–0.3 mm	no record	(12.4–)12.8–13.6–14.3(−15) × (4.2–)4.5–5.1–5.8(−6.8) µm	anthraquinone pigment in apothecia. Spot tests (pigmented portions): K+magenta, KC−, C−, P−, UV−	–	Lendemer et al. (2016)	= *~~~PROTECTED_TN_274~~~ hodgesii*
9	*Synarthonia inconspicua* (Stirt.) Van den Broeck & Ertz	32–46 µm thick, inconspicuous to whitish to greenish-gray, smooth, continuous to cracked	solitary, 0.1–0.5 × 0.1–0.5 mm, rounded; disc heavily white pruinose, light brown when pruina removed, flat to convex	(15–)16.5–21.3(–24.5) × (5.3–)6.2–7.7(–8.5) µm	pycnidia black, walls brown, composed of hyphae of 2.1–2.4 µm wide. Conidia 4.7–6.4 × 0.8–1.3 µm, hyaline	an unidentified xanthone. Spot tests: thallus K−, C−, KC−, PD−, UV+ (often patchily) bright yellow-orange to ± whitish yellow	Broeck et al. (2018)	= *~~~PROTECTED_TN_276~~~ inconspicua*
10	*Synarthonia josephiana* Van den Broeck & Ertz	11 µm thick, whitish, smooth, continuous to cracked	rounded, 0.1–0.4 × 0.1–0.3 mm; disc black, epruinose but with remnants of thallus	15.5–21.5 × 5.5–8.0 µm	pycnidia numerous, black. Conidia 4.4–6.3 × 1.1–1.4 µm, hyaline	without lichen substances. Spot tests: thallus K−, C−, KC−, PD−, UV−	Broeck et al. (2018)	–
11	*Synarthonia karunaratnei* (Weerakoon & Aptroot) Van den Broeck & Ertz	crustose, continuous but often appearing absent, not corticate, dull, pale ochraceous	erumpent to sessile, round or ellipsoidal; disc dark brown to black with an orange hue	8-spored, 9.0–10.5 × 3.5–4.5 µm	–	an anthraquinone. Spot tests: thallus and apothecia UV–, C–, P–, K–	Weerakoon et al. (2016)	= *~~~PROTECTED_TN_279~~~ karunaratnei*
12	*Synarthonia leproidica* Ertz, Aptroot & Diederich	saxicolous, crustose, leproid, forming patches of c. 0.5–5 µm diam.	–	–	–	psoromic acid. Spot tests: thallus K−, C−, PD+ bright orange-yellow, UV−	Ertz et al. (2020)	–
13	*Synarthonia lopingensis* (Zahlbr.) Van den Broeck, Frisch & Ertz	–	0.04–0.9 × 0.04–0.5 mm, numerous; disc black, orange brown and partly translucent when wet, flat	15.0–19.0 × 5.0–6.5 µm	–	without lichen substances. Spot tests: thallus UV+ pale orange, ascomata UV+ dark orange	Broeck et al. (2018)	= *~~~PROTECTED_TN_282~~~ lopingensis*
14	*Synarthonia muriformis* Van den Broeck, Frisch & Ertz	10–19 µm thick, whitish to greenish-gray, smooth to granular, discontinuous,	solitary, 0.1–0.4 × 0.08–0.2 mm, rounded to slightly elongate, never stellate or lirellate; disc heavily white pruinose, black to brown when pruina removed, flat to convex	22.0–36.5 × 10.0–14.5 µm	–	evernic acid, psoromic acid and two unknown UV+ white secondary compounds. Spot tests: thallus K–, C–, KC–, PD+ orange, UV-. Pruina PD+ orange	Broeck et al. (2018)	–
15	*Synarthonia ochracea* (Dufour) Van den Broeck & Ertz	12–18 µm thick, whitish, smooth to cracked; thallus hyphae not observed	solitary, 0.1–0.5 × 0.1–0.5 mm, lirellate, often stellate to lobed, numerous; disc heavily orange pruinose, flat	11.5–17.0 × 4.0–7.5 µm	pycnidia immersed to erumpent, pale, round. Conidia 4.2–5.3 × 0.8–1.3 µm, bacilliform, hyaline, simple	parietin. Spot tests: thallus K+ yellowish, C–, KC–, PD–, UV+ pale orange. Ascomata UV+ dark orange, K+ purplish	Broeck et al. (2018)	= *~~~PROTECTED_TN_285~~~ ochracea*
16	*Synarthonia ochrodes* (Nyl. ex Willey) Van den Broeck & Ertz	–	0.04–0.9 × 0.04–0.5 mm, numerous, young minute; disc reddish brown to black, entirely covered by an orange pruina, convex	13.5–16.5 × 4–6.5 µm	–	without lichen substances. Spot tests: thallus UV+ orange	Broeck et al. (2018)	= *~~~PROTECTED_TN_287~~~ ochrodes*
17	*Synarthonia pilosella* Van den Broeck, Eb. Fisch., Killmann & Ertz	22–33µm thick, green, granular, continuous; thallus hyphae hyaline, 2.7–3.0 µm wide	solitary, rounded, 0.06–0.2 × 0.04–0.2 mm, numerous, sessile; disc heavily white pruinose, orange below the pruina, flat, 1.2–2.9 µm wide	14.5–19.0 × 5.0–7.0 µm	–	unidentified xanthone (major) as in *S. inconspicua* and traces of some other unknown secondary compounds. Spot tests: thallus K– C–, KC–, PD+ yellow, UV–. Pruina PD+ yellow. Ascomata UV+ bright orange-yellow contrasting with the dark UV– thallus	Broeck et al. (2018)	–
18	*Synarthonia psoromica* S. Joseph & G.P. Sinha	corticolous, rimose in appearance, whitish, c. 100 µm thick	rounded to slightly elongated; disc thinly white pruinose	8-spored, (12.5–)13.0–14.7(–15.8) × (4.0–)4.4–5.2(–5.5) µm	–	psoromic acid present. Spot tests: Thallus K–, C–, P+ yellow, UV–	Joseph and Sinha (2015)	–
19	*Synarthonia rimeliicola* (Diederich) Van den Broeck, Diederich & Ertz	–	disc whitish pruinose	–	–	ascomata PD+ yellow	Broeck et al. (2018)	–
20	*Synarthonia robertiana* Soto-Medina & Aptroot	–	–	28–32 × 9–14 µm	–	lichexanthone	Soto (2022)	–
21	*Synarthonia sarcographoides* Aptroot, A.A. Menezes, E.L. Lima & M. Cáceres	crustose, not corticate, usually dull, pale green, very thin and closely following the bark surface	0.1 mm diam., round, black, white pruinose	8-spored, 20–22 × 11.0–12.5 µm	–	three unidentified xanthones. Spot tests: thallus and ascomata UV–, C–, P–, K–	Menezes et al. (2013)	–
22	*Synarthonia sikkimensis* S. Joseph & G.P. Sinha	sorediate, corticolous, crustose, rimose-like, smooth to verrucose, 100–180 µm thick	elongated to lirellate, with a thin white margin; disc thinly white pruinose	8-spored, (17.0–)19.0–23.0(–25.7) × (5.5–)6.5–7.5(–8.6) µm	–	no substances detected. Spot tests: thallus K–, C–, P–, UV–	Joseph and Sinha (2015)	–
23	*Synarthonia stigmatidialis* Müll. Arg.	corticolous, rimose, whitish	with an inconspicuous to thin white margin, individual ascomata rounded to short lirellate; disc thinly white pruinose	8-spored, (12.0–)14.5–19.0(–22.0) × (4.8–)5.4–6.6(–7.0) µm	–	no substances detected. Spot tests: thallus K–, C–, P–, UV–	Joseph and Sinha (2015)	–
24	*Synarthonia xanthonica* Aptroot	crustose, continuous, not corticate, dull, pale ochraceous white	solitary, irregularly linear to ink spot-like branched in outline, 0.3–0.8 mm wide; disc dark gray, white pruinose	8-spored, 12–13.5 × 4.5–5.5 µm	–	a xanthone, probably 1,8-dihydroxy-3,6-dimethoxyxanthone. Spot tests: thallus UV+ orange, C–, K–, KC–, P–	Aptroot et al. (2022)	–
25	*Synarthonia xanthosarcographoides* Aptroot	crustose, corticolous, mineral gray, dull, without prothallus	punctiform to inkspot-like, eventually confluent, 0.2–0.4 mm diam.; disc bluish gray, dull; margin whitish	8-spored, 20–22 × 8–9 µm	–	lichexanthone. Spot tests: thallus UV–, C–, P–, K–; pseudostromata UV+ yellow	Aptroot and Spielmann (2020)	–

The genus *Chicitaea* presently comprises four accepted species (Table 3). In comparison with these species, our specimen exhibits distinctive features, including lacking soredia and pycnidia, containing an unknown lichen substance distinct from perlatolic acid and 2’-O-menthylperlatolic acid. Phylogenetic analyses further support its placement as a distinct lineage within *Chicitaea*, providing robust evidence for the recognition of our collection as a new species. According to the current phylogenetic tree, *C. confusa* and *C. lecanoriformis* exhibit a close genetic distance, which has led to proposals for their taxonomic merging. However, we contend that this conclusion is premature, as only a mtSSU sequence is available for *C. lecanoriformis*, and comparative analysis of additional gene regions is critical for robust taxonomic resolution.

Through a review of the taxonomic literature on genus *Coniocarpon*, we compiled morphological descriptions for approximately 23 effective species (Table 4). While databases (Index Fungorum and MycoBank) indicate that about 12 species have been synonymized and transferred to other genera. The 23 species were categorized into two groups based on the availability of ascospores data: one with comprehensive ascospores measurements (11) and another lacking such data (12). Among the 11 species for which ascospores’ data were available, they were divided into two groups based on whether their ascospores length is ≥ 21 μm: five species have ascospores ≥ 21 μm. The species under study in this work falls into this category. The five species (*Coniocarpon carneoumbrinum*, *Coniocarpon cinnabarinum*, *Coniocarpon fallax*, *Coniocarpon rubrocinctum*, *Coniocarpon tuckermanianum*) all possess pruinose disc, while the disc of *Coniocarpon chishuiense* lacks this characteristic. The thallus of Species *Coniocarpon carneoumbrinum*, *Coniocarpon fallax*, and *Coniocarpon tuckermanianum* respectively are gray, pale brown and white, while the new species has an olive thallus. Chemically, *Coniocarpon cinnabarinum* and *Coniocarpon rubrocinctum* contain only psoromic acid, while *C. chishuiense* contains an unknown lichen substance in addition to psoromic acid.

Although the lack of ascospores data in some comparative species precludes a comprehensive comparison, the morphological differences in their thalli and apothecia suffice for effective differentiation. The thallus color distinguishes the eight species from *C. chishuiense* (white *vs*. olive). The ascomata of both *Coniocarpon nigrum* DC. and *Coniocarpon olivaceum* DC. are pruinose, while those of *C. chishuiense* are not. Different from species *Coniocarpon subrotundum* (Fée) R. Meissn., *Coniocarpon torulosum* (Fée) Fée and *Coniocarpon vulgare* Rabenh, which have black ascomata, *C. chishuiense* has brown ascomata. Compared to the other three species, the characters of *C. chishuiense* are: olive, non-powdery, rough thallus, and brown, rounded ascomata. This combination of features clearly distinguishes it from *Coniocarpon cascarillae* Fée (smooth thallus), *Coniocarpon gracile* (Eschw.) A. Massal. (wine-red, linear ascomata), and *Coniocarpon tricolor* (Ach.) Duby (powdery thallus, rufous ascomata). The new species can be distinguished from *Coniocarpon ferrugineum* Delise and *Coniocarpon xanthostigma* (Ach.) Duby primarily by its apothecial morphology and color: the new species has brown, convex, and epruinose apothecia; *C. ferrugineum* possesses reddish-brown, sorediate-form apothecia; while *C. xanthostigma* exhibits yellowish-green, flat, and powdery apothecia. The powdery, gray to brown thallus of *Coniocarpon caribaeum* Fée and the non-lichenized thallus with black apothecia of *Coniocarpon sphaerale* Duby both differ from those of *C. chishuiense*.

*Synarthonia* currently comprises about 25 recognized species (Table 5), which can be divided into two morphological groups based on the presence or absence of lichen substance. Our newly reported record falls within the group lacking lichen substances, comprising eight species. Among these, only two species show white pruina on the apothecial disc, a trait also present in our specimen. Based on the above analyses, our collection is most similar to *S. sikkimensis* and *S. stigmatidialis*. However, it can be readily distinguished from *S. sikkimensis*, which has a sorediate thallus and an I+ reddish hymenial reaction (*vs*. I+ blue in our specimen). In comparison to *S. stigmatidialis*, our collection lacks the rimose thallus and also differs in hymenium reaction (I+ reddish *vs*. I+ blue). In the phylogenetic analysis, the new collection forms a strongly supported clade with *S. inconspicua*. Based on this evidence, we identify our collection as *Synarthonia inconspicua*, representing a new record for China.

## Supplementary Material

XML Treatment for Chicitaea yueliangshanensis

XML Treatment for Coniocarpon chishuiense

XML Treatment for Synarthonia inconspicua

## Additional information

### Use of AI

No use of AI was reported.

### Author ORCIDs

Lin-Zhi He https://orcid.org/0009-0008-3621-7339

Wei Wu https://orcid.org/0009-0009-1191-6667

He-Yun Bo https://orcid.org/0009-0003-8641-1619

Lin-Shan Chai https://orcid.org/0009-0005-2458-0454

Ruvishika S. Jayawardena https://orcid.org/0000-0001-7702-4885

Sheng Liang https://orcid.org/0009-0002-4376-568X

Qing-Feng Meng https://orcid.org/0000-0001-9814-8238

Shao-Bin Fu https://orcid.org/0000-0001-9932-1346

### Data availability

All of the data that support the findings of this study are available in the main.
